# COGNITIVE IMPAIRMENT AND SOCIAL DETERMINANTS OF HEALTH AMONG INDIGENOUS WOMEN

**DOI:** 10.1093/geroni/igae098.2968

**Published:** 2024-12-31

**Authors:** Soonhee Roh, Yeon-Shim Lee, Heehyul Moon, Donald K Warne, Serene Thin-Elk, Sasheen T Stone

**Affiliations:** University of South Dakota, Sioux Falls, South Dakota, United States; San Francisco State University, San Francisco, California, United States; University of Louisville, Louisville, Kentucky, United States; Johns Hopkins University, Baltimore, Maryland, United States; South Dakota Urban Indian Health, Sioux Falls, South Dakota, United States; Yankton Sioux Tribe, Wagner, South Dakota, United States

## Abstract

The current study aimed to examine 1) differences between cognitively impaired and cognitively intact groups regarding demographics, education, health literacy, social and community involvement (i.e., social engagement and religious activity/participation), and health (i.e., chronic conditions), 2) the amount to Alzheimer’s Disease and Related Dementias (ADRD) knowledge among the cognitively impaired group, and 3) predictors of cognitive impairment. We considered three dimensions of social determinants of health (SDOH) to predict cognitive impairment: education and health literacy, social and community involvement, and health (i.e., depression and chronic conditions). Collaborating with a Northern Plains tribe, participants were recruited 123 self-identified Indigenous women aged 40 to 70 through a comprehensive recruitment strategy. Employing the SDOH framework, the research assessed cognitive impairment and Alzheimer’s disease knowledge, utilizing the Ascertain Dementia 8 and AD knowledge scales (ADK-30). The investigation examined the relationships between selected SDOH variables and cognitive impairment status. More than half of the participants showed signs of cognitive impairment, which correlated with lower income and education levels. Increased knowledge about Alzheimer’s disease, particularly in terms of treatment management and its life impact subscales, was associated with lower odds of cognitive impairment. Conversely, higher levels of depressive symptoms and participation in religious activities were linked to increased odds of cognitive impairment. Findings underscore the importance of culturally grounded tools and SDOH frameworks tailored to Indigenous contexts in addressing ADRD disparities. Future research should integrate historical and cultural factors to advance health equity within Indigenous communities, ultimately mitigating the impact of ADRD and promoting overall well-being.

